# Artificial Intelligence for Ultrasound Informative Image Selection of Metacarpal Head Cartilage. A Pilot Study

**DOI:** 10.3389/fmed.2021.589197

**Published:** 2021-03-01

**Authors:** Edoardo Cipolletta, Maria Chiara Fiorentino, Sara Moccia, Irene Guidotti, Walter Grassi, Emilio Filippucci, Emanuele Frontoni

**Affiliations:** ^1^Rheumatology Unit, Department of Clinical and Molecular Sciences, Polytechnic University of Marche, Ancona, Italy; ^2^Department of Information Engineering, Polytechnic University of Marche, Ancona, Italy; ^3^Department of Advanced Robotics, Italian Institute of Technology, Genoa, Italy

**Keywords:** hyaline cartilage, ultrasonography, metacarpal head, artificial intelligence, deep learning, convolutional neural network, rheumatoid arthritis, osteoarthritis

## Abstract

**Objectives:** This study aims to develop an automatic deep-learning algorithm, which is based on Convolutional Neural Networks (CNNs), for ultrasound informative-image selection of hyaline cartilage at metacarpal head level. The algorithm performance and that of three beginner sonographers were compared with an expert assessment, which was considered the gold standard.

**Methods:** The study was divided into two steps. In the first one, an automatic deep-learning algorithm for image selection was developed using 1,600 ultrasound (US) images of the metacarpal head cartilage (MHC) acquired in 40 healthy subjects using a very high-frequency probe (up to 22 MHz). The algorithm task was to identify US images defined informative as they show enough information to fulfill the Outcome Measure in Rheumatology US definition of healthy hyaline cartilage. The algorithm relied on VGG16 CNN, which was fine-tuned to classify US images in informative and non-informative ones. A repeated leave-four-subject out cross-validation was performed using the expert sonographer assessment as gold-standard. In the second step, the expert assessed the algorithm and the beginner sonographers' ability to obtain US informative images of the MHC.

**Results:** The VGG16 CNN showed excellent performance in the first step, with a mean area (AUC) under the receiver operating characteristic curve, computed among the 10 models obtained from cross-validation, of 0.99 ± 0.01. The model that reached the best AUC on the testing set, which we named “MHC identifier 1,” was then evaluated by the expert sonographer. The agreement between the algorithm, and the expert sonographer was almost perfect [Cohen's kappa: 0.84 (95% confidence interval: 0.71–0.98)], whereas the agreement between the expert and the beginner sonographers using conventional assessment was moderate [Cohen's kappa: 0.63 (95% confidence interval: 0.49–0.76)]. The conventional obtainment of US images by beginner sonographers required 6.0 ± 1.0 min, whereas US videoclip acquisition by a beginner sonographer lasted only 2.0 ± 0.8 min.

**Conclusion:** This study paves the way for the automatic identification of informative US images for assessing MHC. This may redefine the US reliability in the evaluation of MHC integrity, especially in terms of intrareader reliability and may support beginner sonographers during US training.

## Introduction

Hyaline cartilage is a highly specialized connective tissue characteristic of synovial joints. Its principal function is to provide low-friction articular surfaces and to act as a shock absorber during the joint movement (1). Hyaline cartilage lacks blood vessels, thus it has a limited capacity for intrinsic healing and repair. In this regard, the integrity of this noble tissue is essential to joint health. The chondrocyte, the unique cell type in adult hyaline cartilage, maintains a stable equilibrium between the synthesis and the degradation of extracellular matrix components. With age and/or in the presence of various rheumatic diseases, such as rheumatoid arthritis and osteoarthritis, this balance is undermined, and the catabolic activity exceeds the anabolic one, thus leading to dehydration, degeneration, and thinning of the cartilage layer (2).

Although conventional radiography is the most adopted imaging method for the assessment of joint damage in daily clinical practice, it provides only an indirect visualization of the hyaline cartilage through the evaluation of the joint space narrowing. The accuracy of conventional radiography has been questioned in non-weight-bearing joints such as the ones of hands and wrists, which are commonly involved in different rheumatic diseases (3, 4). Moreover, in several studies, conventional radiography was found to be less sensitive than ultrasonography (US) in the detection of joint damage (5–11).

Recently, US has been suggested as a reliable and reproducible tool for the assessment of the hyaline cartilage of the small joints of the hand (4, 11–17). One of the main drawbacks of US is its subjectivity in the interpretation of US findings and the consequent variable inter- and intraobserver reliability (18–20). This issue is particularly relevant for the beginner musculoskeletal sonographer (18–20). Thus, the development of a tool that can enhance the US learning process is noteworthy (21).

In the last few years, artificial intelligence has been gaining importance in US, and a number of advantages have been claimed for this alternative method over the conventional acquisition and interpretation of US images, including faster performance, higher reliability and better standardization of image acquisition (22–27). Only a few studies have applied artificial intelligence in the field of musculoskeletal diseases (28–32), and no studies explored the artificial intelligence (AI) in the US assessment of hyaline cartilage, except our previous preliminary work (33).

To date, deep learning (DL) has shown its value in the healthcare domain for computer-assisted medical image analysis (24). DL is a branch of AI, and its algorithms are inspired by human brain, being able to learn from a large amount of data by itself. In fact, DL has the advantage of directly learning image features from raw data, avoiding the need to design hand-crafted features as for traditional machine learning approaches (23). In particular, Convolutional Neural Networks (CNNs), one of the most popular DL algorithms, are widely used for medical image analysis tasks such as classification, segmentation, detection, and biometric measurements, with applications in diagnosis and image-guided interventions and therapy (23). CNNs are able to recognize complex structures in an image by applying convolutional filters (each defined by a kernel matrix) whose weights are iteratively learned during CNN training. Training a CNN relies on labeled data, which are used to learn the mapping from the input to the desired output. Training a CNN from scratch requires a large amount of labeled data, which may not always be available, especially in the medical image analysis domain where image labeling by expert clinicians is a resource-expensive procedure. Moreover, training a CNN from scratch may often lead to overfitting issues (i.e., CNN inability to generalize on new sets of data). A feasible alternative is to exploit transfer learning. Transfer learning consists of extracting knowledge from one task (where large annotated datasets are available) and using the extracted knowledge for a second one. It has been demonstrated that using transfer learning is particularly useful for medical image analysis since limited training data are usually available (34, 35).

Driven by this last consideration, we decided to use transfer learning for training a CNN for US informative-frame selection. We investigated CNNs pretrained on ImageNet, a large image database that includes more than 1 million of annotated natural images (e.g., cats, dogs, cars).

The main aim of this study was to develop an automatic DL algorithm for US informative-image selection of hyaline cartilage at metacarpal head level. An image was defined informative when it shows enough information to fulfill the Outcome Measure in Rheumatology US definition of healthy hyaline cartilage (36). The algorithm performance and that of three beginner sonographers were compared with an expert assessment, which was considered the gold standard.

## Materials and Methods

### Study Design

The study was conducted from January 2019 to March 2020. The study was divided in two steps. In the first one, a CNN algorithm for informative image selection was developed and trained using 1,600 static US images. The US images were acquired by an expert (E.F.) in musculoskeletal US who evaluated the metacarpal head cartilage (MHC) from the 2nd to the 5th digit bilaterally in 40 healthy subjects.

In the second step of the study, the CNN output was compared with the conventional assessment of the MHC carried out independently by three beginner (E.M., F.F., and J.D.B.) and the expert (E.F.) sonographers. A beginner sonographer was defined as a sonographer with limited experience (<1 year) in the US assessment of hyaline cartilage. MHC from the 2nd to the 5th digit of both hands of eight healthy subjects was independently evaluated on the same day by the beginner and the expert sonographers. The beginner sonographers were asked to provide a set of eight US images per subject (one US image per each metacarpal head) for a total of 192 static images and a set of eight 10-s videoclips on the same healthy subjects for a total of 192 videoclips. A random sample of 128 static images and 64 videoclips were used in the reliability analysis. The US images evaluated by the beginners (each beginner judged a third of the US images) and the videoclips assessed by the algorithm were tested against the expert opinion who had to state whether the US images were informative or not. Expert was blinded to the US images authorship (i.e., beginner sonographer or artificial intelligence algorithm). The time required for each US examination was registered.

### Subjects

Healthy subjects were selected from relatives visiting or accompanying in- and out-patients, friends of the authors, and medical students attending the “Carlo Urbani” Hospital (Jesi, Ancona, Italy). Healthy subjects were enrolled because they had the lowest probability to present US abnormalities indicative of cartilage damage. In fact, pathologic findings may generate a bias in the interpretation of US images by both the beginner sonographers and the algorithm.

Exclusion criteria were as follows: (i) previous diagnosis of inflammatory/degenerative arthropathies; (ii) joint pain [visual analog scale (VAS) ≥10/100] and/or analgesic or non-steroidal anti-inflammatory drugs' intake in the 4 weeks preceding the visit; (iii) age <18 years old; and (iv) hard tissue enlargement or deformity of the metacarpophalangeal, proximal, or distal interphalangeal joints suggestive of hand osteoarthritis and/or joint inflammation at physical examination. The following demographic data were recorded: sex, age, handedness, height, weight, and body mass index.

### US Image Acquisition and Interpretation

US examinations were carried out using a MyLab Class C (Esaote SpA, Genoa, Italy), equipped with a very high-frequency broadband linear probe (10–22 MHz). A grayscale standard setting was adopted (B-mode frequency: 22 MHz, master gain: 70%, mechanical index: 0.3, dynamic range: 12, depth 15 mm, focus position at the area of interest).

The hands were placed on the table, with the metacarpophalangeal joints in maximal flexion (>60°) (14, 36, 37). The metacarpal head from the 2nd to the 5th digit of both hands was scanned on the dorsal aspect from radial to ulnar and from proximal to distal sides to ensure the maximal exploration of the hyaline cartilage using the EULAR standard scans (38). Particular attention was paid to ensure a perpendicular insonation of the cartilage surface (14, 36, 37).

**Figure 1:**
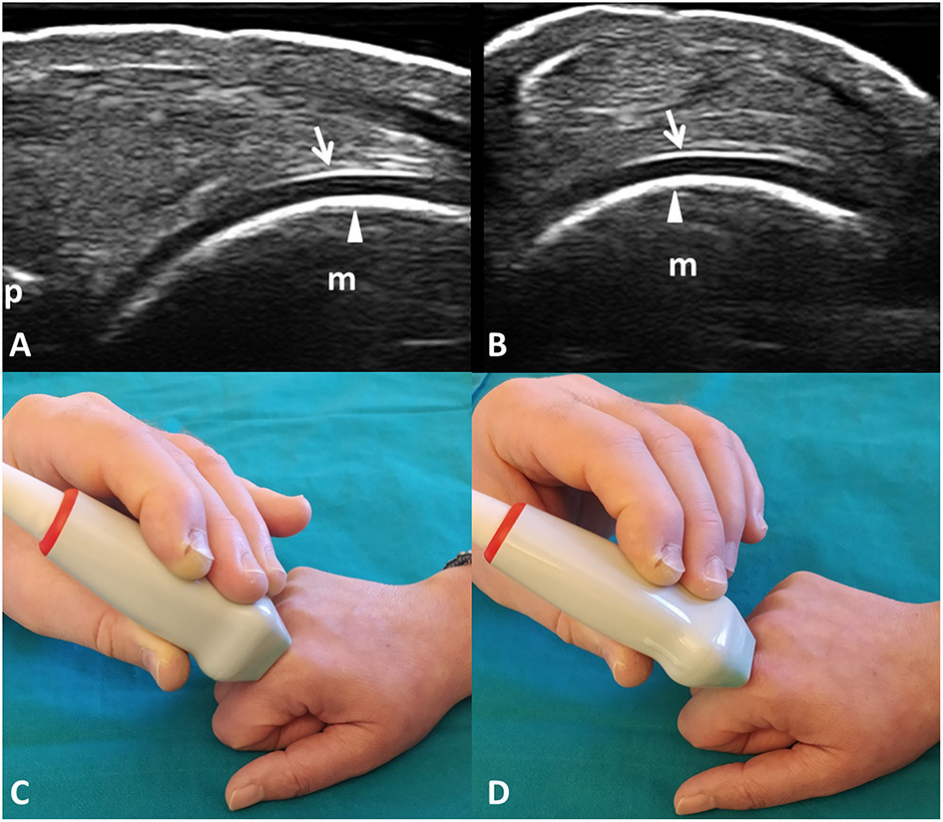
Healthy hyaline cartilage. Dorsal longitudinal **(A)** and transverse **(B)** scans of the hyaline cartilage of the 2nd metacarpal head in a healthy subject. Hyaline cartilage appears as a homogeneously hypo-anechoic layer delimited by two regular, sharp, continuous, and hyperechoic interfaces where insonated orthogonally. Arrows indicate the outer margin (i.e., the chondrosynovial interface); arrowheads indicate the inner margin (i.e., the osteochondral interface). **(C,D)** The position of the probe in the dorsal longitudinal **(C)** and transverse **(D)** scans. m, metacarpal head; p, base of the proximal phalanx.

The normal appearance of the hyaline cartilage is characterized by a homogenous hypo-anechoic layer delimited by two regular, sharp, and bright margins where insonated orthogonally (36, 37). An US image displaying such characteristics was defined informative. The detection in the videoclip by the algorithm or by the expert of at least a frame showing US features of healthy hyaline cartilage was a sufficient criterion to classify it as informative. Figure 1 provides a pictorial evidence of a healthy MHC.

### CNN Algorithm for Informative Frame Selection

A VGG16 CNN, pre-trained on the ImageNet dataset, was used for transfer learning. The VGG16 architecture was chosen for two main reasons: (1) its sequential architecture results to be particularly suitable for small-size training set and low image variability (2, 39) its shallow architecture (only 3 × 3 convolutional layers stacked on top of each other in increasing depth) is associated with low computation cost.

In the original VGG16 architecture implementation, the ImageNet images are processed through 13 convolutional (conv) layers to perform feature extraction. The filters used in each conv layer have very small receptive field (3 × 3) (the smallest size to capture notion about left/right, up/down and center), followed by a rectified linear unit (ReLU) activation function. After the convolutional layers, three fully connected (FC) layers (4,096 neurons in the first two layers and 1,000 neurons in the last one) followed by a softmax layer are used to predict class probability.

**Figure 2:**
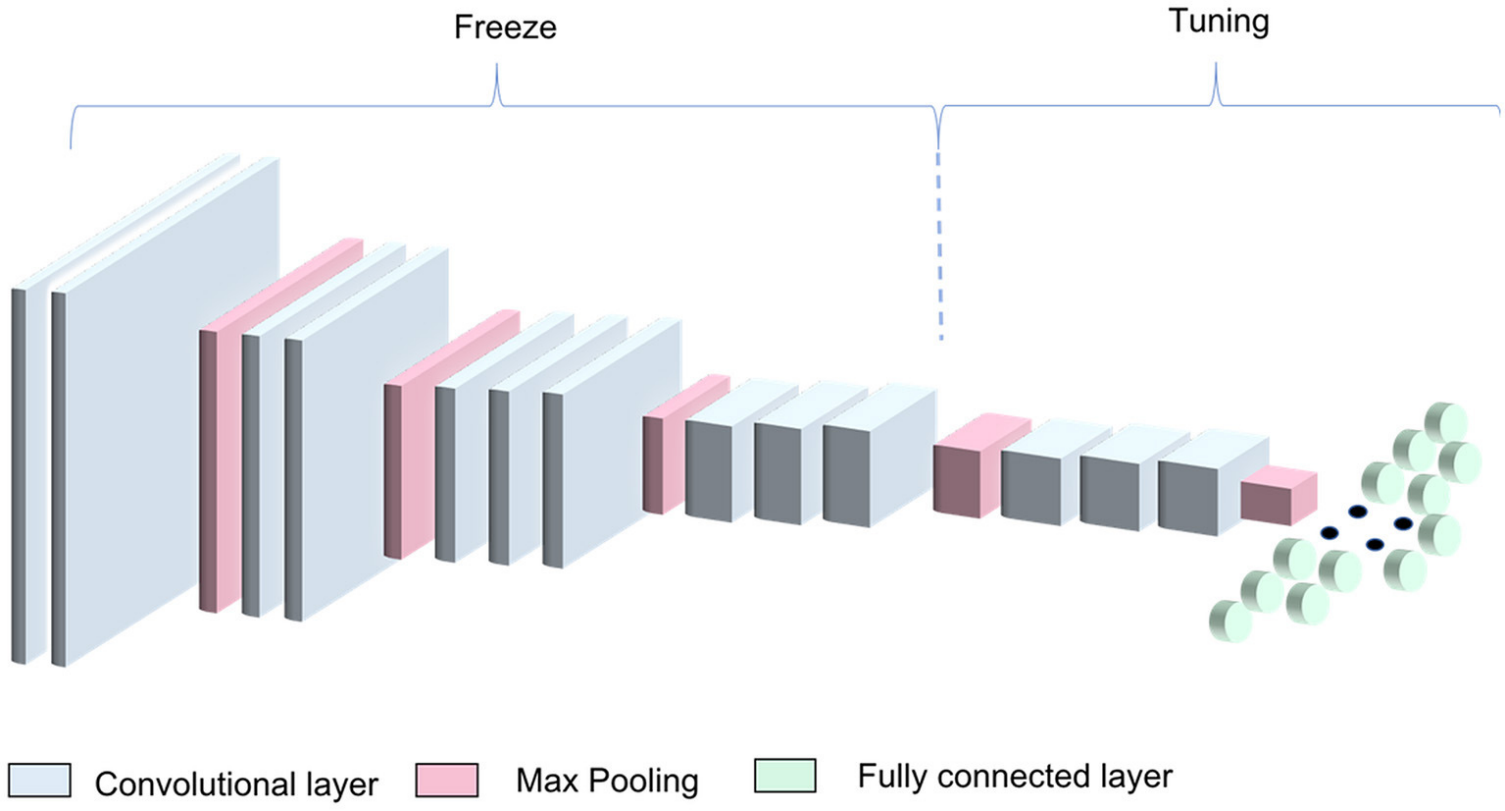
VGG16 transfer-learning strategy. The algorithm was obtained by freezing the first four blocks of the VGG16 pretrained on the ImageNet dataset and training the remaining blocks along with the dense layers.

In this work, the ImageNet pretrained weights were used as a starting point for the CNN training process. We modified the three FC layers using 1,024, 512, and 2 neurons in the first, second, and third FC layers, respectively, to adapt the architecture to our binary classification problem (informative—non-informative image classification) (Figure 2). The architecture was trained freezing the first 10 conv layers and tuning the remaining ones. In this way, we managed to exploit the knowledge encoded in the VGG16 trained on the large ImageNet dataset.

### Training Strategy

Prior training the VGG16, US images were resized to 224 × 224 pixels and converted to RGB images, repeating the grayscale channel for three times, to match the image input dimension required by the pre-trained VGG16. Intensity mean was removed from each image.

The mini-batch gradient descent, with a momentum of 0.9, was used as optimizer using the categorical cross-entropy as loss function. The batch size was set to 64 as a balance between training speed and gradient convergence. Training was performed for 100 epochs with a learning rate of 0.0001.

A leave-four-subject out cross-validation was performed for testing the classification. The dataset was divided into 10 subsets of subjects. In turn, one of the 10 subsets (containing four subjects) was used as the test set while the other nine subsets were used as training set. The validation was performed selecting randomly four subjects from the training set, obtaining in such a way 10 models.

The analyses were performed using Keras with TensorFlow library as backend on Google Colaboratory (https://colab.research.google.com/).

### Performance Metrics

To measure the performance of our approach, we computed the mean area under the curve (AUC) of the operating characteristic curve (ROC) and the mean classification Precision (*Prec*), Recall (*Rec*), and f1-score (*f1*) for the *i*th class, with i ∈ C (informative, non-informative), where *TP*_*i*_,*FP*_*i*_,*FN*_*i*_ were the true positives, false positives, and the false negatives, respectively.

(1) $$\begin{array}{l} Prec_{i}=\frac{TP_{i}}{TP_{i}\;+\;FP_{i}} \end{array}$$

(2) $$\begin{array}{l} Rec_{i}=\frac{TP_{i}}{TP_{i}\;+\;FN_{i}} \end{array}$$

(3) $$\begin{array}{l} f1_{i}=\frac{2\;x\;Prec_{i}\;x\;Rec_{i}}{Prec_{i}\;+\;Rec_{i}} \end{array}$$

### Statistical Analysis

Results are expressed as number and/or corresponding percentage for qualitative variables and as mean and standard deviation (SD) for quantitative variables. The Chi-square test and the Mann-Whitney test were used to compare the qualitative and quantitative variables, respectively. The agreement in the informative image selection between the expert (i.e., the gold standard) and the algorithm, and between the expert and the conventional assessment of the beginners was calculated using an unweighted Cohen's kappa and interpreted according to Landis and Koch (40).

Two-tailed *p*-values < 0.05 were considered significant. Statistical analysis was performed using Statistical Package for the Social Sciences (SPSS) software (version 26.0 for Windows, Chicago, Illinois, USA).

## Results

### Subjects

A total of 48 healthy subjects were included in this monocentric and cross-sectional study: 40 in the training and testing of the algorithm (first step) and 8 in the reliability analysis (second step). Table 1 shows the main demographic characteristics of the participants.

**Table 1:** Demographic characteristics of the healthy participants.

Sex (female/male)	33/15
Age (years, mean ± SD)	54.6 ± 5.6
Handedness (right/left)	40/8
Height (cm, mean ± SD)	170.7 ± 9.5
Weight (kg, mean ± SD)	72.4 ± 6.4
Body mass index (kg/m^2^, mean ± SD)	24.8

*SD, standard deviation*.

### Artificial-Intelligence Algorithm: Training and Testing

**Figure 3:**
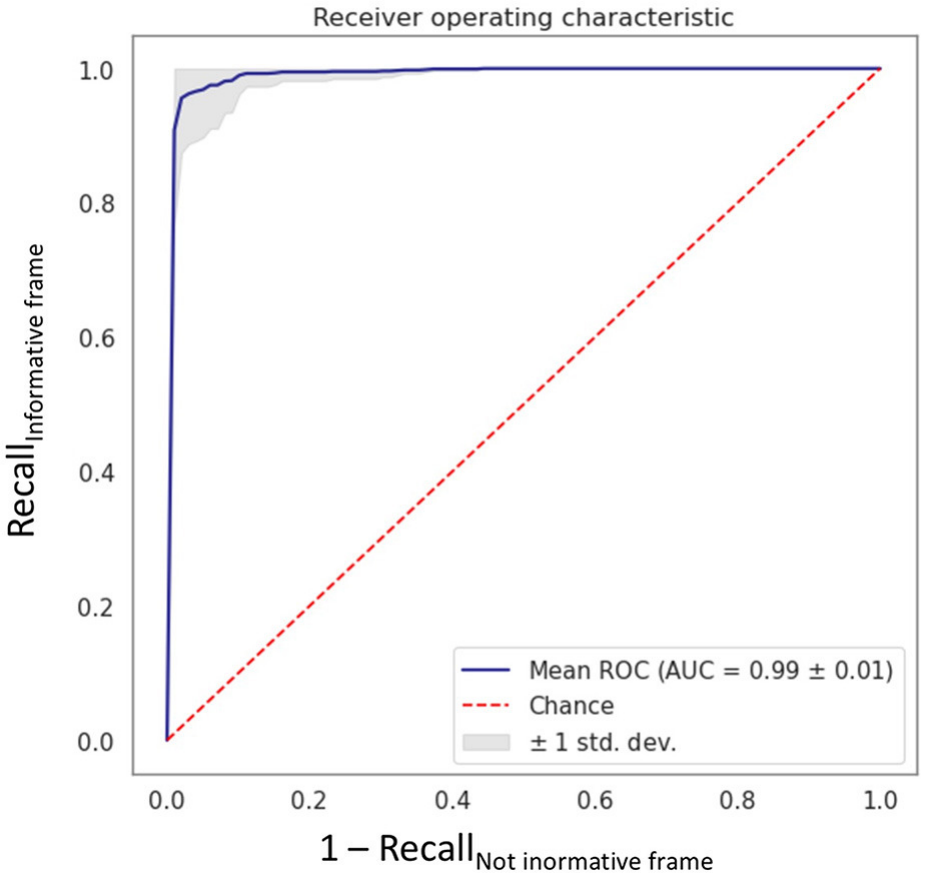
Receiving operating characteristic curve obtained with the fine-tuned VGG16 CNN algorithm “MHC identifier 1.” AUC, area under the curve; ROC, receiving operating characteristics; std dev, standard deviation.

In the first step of the study, the VGG16 CNN showed excellent performance in the informative image selection task, with an AUC of 0.99 ± 0.01 (Figure 3) computed among the 10 models.

Table 2 shows the classification results for both informative and non-informative frame obtained from the cross-validation procedure.

**Table 2:** Classification metrics for precision, recall, and f1-score, with i ∈ C (informative, non-informative).

	***i***	**Precision**	**Recall**	**f1-score**
VGG16 CNN algorithm “MHC identifier 1”	Informative	0.94 (0.07)	0.98 (0.02)	0.97 (0.05)
	Non-informative	0.98 (0.02)	0.96 (0.06)	0.97 (0.04)

Values in brackets are the standard deviation.

*MHC, metacarpal head cartilage*.

**Figure 4:**
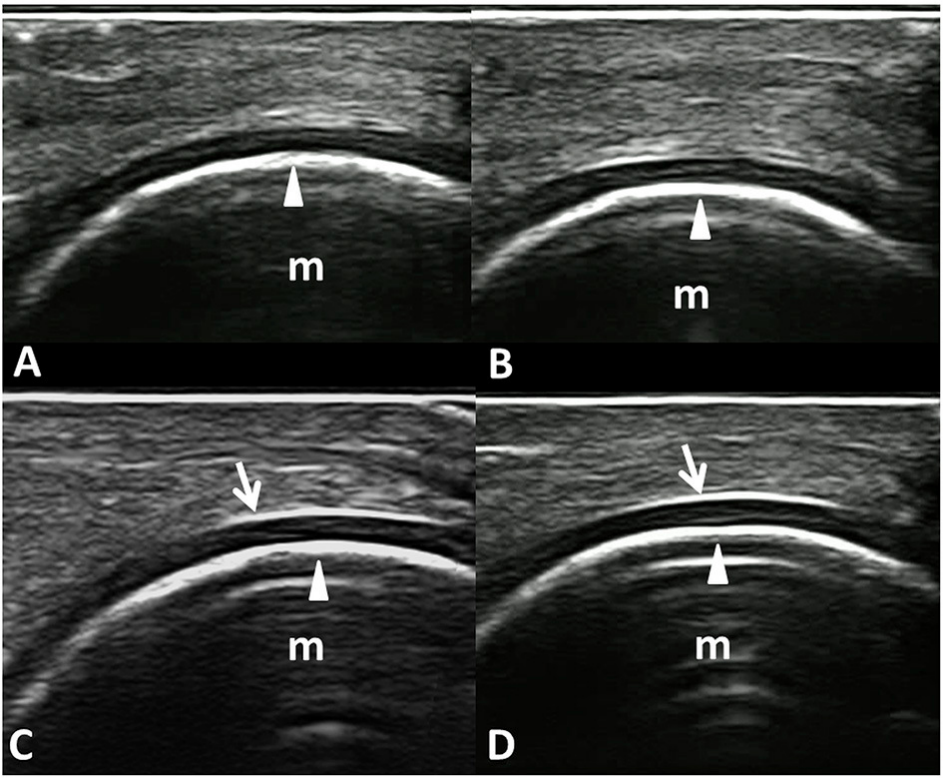
Informative and non-informative US frame selected by the VGG16 CNN algorithm “MHC identifier 1.” Dorsal longitudinal scans acquired at the metacarpal head level in healthy subjects. **(A,B)** Examples of non-informative frames; **(C,D)** Examples of informative frames. In fact, while the inner margin (arrowheads) is evident in all the panels, the chondrosynovial (arrows) interface is clearly visible only in the lower images **(C,D)**. m, metacarpal head; arrows, chondrosynovial interface; arrowheads, osteochondral interface.

Figure 4 shows an example of informative and non-informative frames selected by the model that reached the best AUC, which we named “MHC identifier 1.”

### Feasibility

The average time required to complete the conventional US assessment was 6.0 ± 1.0 and 4.0 ± 0.5 min for beginners and expert sonographer, respectively (*p* < 0.01). On the other hand, the time spent to acquire the videoclips was 2.0 ± 0.8.

### Reliability Analysis

The agreement between the automatic algorithm and the expert sonographer was almost perfect (Cohen's kappa: 0.84, 95% confidence interval: 0.71–0.98); whereas, the agreement between the expert and the beginners using conventional assessment was moderate (Cohen's kappa: 0.63, 95% confidence interval: 0.49–0.76) (*p* < 0.01) (Table 3) without significant difference in the interobserver agreement between the expert and each beginner sonographer (*p* = 0.14).

**Table 3:** Interobserver agreement.

	**Beginners**		
**US static images (*****n*** **= 128)**		**Informative**	**Non-informative**
Expert	Informative	56 (43.8)	8 (6.3)
	Non-informative	16 (12.5)	48 (37.5)
	**Total agreement:** 81.3%		
	**MHC Identifier 1**		
**10-s videoclips (*****n*** **= 64)**		**Informative**	**Non-informative**
Expert	Informative	31 (48.4)	2 (3.1)
	Non-informative	3 (4.7)	28 (43.8)
	**Total agreement:** 92.2%		

Values in brackets are percentages.

MHC, metacarpal head cartilage; US, ultrasound.

*The beginner sonographers and the VGG16 CNN algorithm “MHC identifier 1” agreement with the expert sonographer considered the gold standard*.

## Discussion

Last-generation US systems allow the real-time identification of otherwise undetectable musculoskeletal abnormalities, which have a growing impact in the management of many rheumatic diseases (41, 42). However, US is a highly operator-dependent technique, and sonographer skills and experience may affect both acquisition and interpretation processes (19, 20). Several international initiatives were undertaken to ensure the standardization of US assessment and to increase its reproducibility in rheumatological setting (36, 38, 43, 44). The use of artificial intelligence in musculoskeletal US may further increase its reproducibility and may save sonographers time as shown in cardiological setting (45).

The correct acquisition of an US image is the essential step to ensure an accurate and reliable assessment of the image itself (31, 39, 46). Thus, we believe that the availability of an algorithm facilitating the identification of the region of interest during the acquisition process of US images represents a further step toward the standardization of US examination.

This study describes the first steps taken to develop an algorithm that can identify informative US images for the assessment of the MHC. The application of such an algorithm, that we called “MHC identifier 1,” may redefine the US reliability in the evaluation of the MHC integrity, especially in terms of intrareader reproducibility. MHC identifier 1 showed an almost perfect agreement with the expert sonographer. Here are some possible explanations of disagreement between the algorithm and the expert: while processing an US videoclip, the algorithm may detect even just one frame to define it as informative, which may be missed or considered not relevant by the expert assessment. Conversely, the expert may consider sufficiently assessable US images even if rejected by the algorithm for not strictly fulfilling all the morphostructural criteria. The choice to use both videoclips and static images could be considered a limit of the present study. However, we decided to test the performance of the algorithm using videoclips instead of pictures, since its task will be to support the sonographer during a real-time US examination and not only in the interpretation of static images. Conversely, the sonographer selects a representative US image which conveys the message of a part of the US examination. Finally, according to the data we recorded, the use of this algorithm may shorten the time required to obtain informative US images up to one-third. However, it should be borne in mind that, to date, MHC identifier 1 cannot be routinely applied to clinical practice. In fact, the images must be manually exported from the US system and transferred in a computer where the algorithm can analyze them. A future development may include the incorporation of MHC identifier1 into an US machine to test the clinical value and the feasibility of this method in real-life setting. Thus, the feasibility of this method, including easiness of its use, costs, and availability are yet to be determined.

Our study presents some limitations. First, the same expert sonographer, who served as the imaging “gold standard,” was also the teacher and tutor of the beginner sonographers. This fact may imply a possible risk of systematic bias. Second, only a relatively low number of US images were used in the reliability exercise. However, this limit should be read in light of the fact that it is a pilot study. Third, its monocentric design may limit the generalizability of our results. Fourth, the impact of machine-assisted acquisition of US images was not evaluated. Furthermore, the intrareader reliability of both the sonographers and the algorithm was not tested. Finally, the impact of using different US systems needs to be tested, as images in this study were all obtained with the same US system.

The assessment of MHC status is progressively gaining a relevant role in the management of patients with different chronic arthropathies. In fact, in 2019, the Outcome Measure in Rheumatology US Working Group proposed the US definitions of cartilage damage in patients with rheumatoid arthritis and osteoarthritis (36, 44). The same group of experts is currently carrying out a Delphi exercise to define and quantify the structural joint damage (including cartilage lesion) in rheumatoid arthritis by US.

Although preliminary, our results open up new horizons in the use of artificial intelligence in the US evaluation of hyaline cartilage. In fact, the algorithm MHC identifier 1 could enhance the learning process improving the awareness of the beginner sonographer regarding the probe positioning required to obtain images conveying essential information to assess the MHC. In addition, the ability of this algorithm to identify informative frame on videoclips may suggest its use as a tool that could assist the sonographer during the real time US examination.

Further implementation may allow to measure the MHC thickness and to evaluate the hyaline cartilage of other sites commonly involved by rheumatic diseases such as the knee and the hip (16, 17, 47).

The possibility of identifying, evaluating, and measuring the hyaline cartilage in a reliable and faster way may reduce the US examination time, shorten the learning curve of beginner sonographers by taking advantage of AI feedbacks, and promote new studies in this field (e.g., aimed to compare the semiquantitative scoring system of cartilage damage and the quantitative assessment, and to follow-up the progression of cartilage damage).

In conclusion, this study describes the first steps in the development of an algorithm identifying informative US images for assessing the MHC. The automatic selection of US images acquired by beginner sonographers resulted reliable and feasible, as shown by the comparison with an expert sonographer. The application of such an algorithm may redefine the US reliability in the evaluation of the MHC integrity, especially in terms of intrareader reliability and may support beginner sonographers during US training. However, this algorithm needs further validation before its use in clinical practice.

## Data Availability Statement

The raw data supporting the conclusions of this article will be made available by the authors, without undue reservation.

## Ethics Statement

Ethical review and approval was not required for the study on human participants in accordance with the local legislation and institutional requirements. The patients/participants provided their written informed consent to participate in this study.

## Author Contributions

EC, EFi, MF, and SM substantially contributed to study conception and design, acquisition, analysis, and interpretation of data. IG substantially contributed to acquisition, analysis, and interpretation of data. EFr and WG substantially contributed to analysis and interpretation of data. All the authors revised the paper and approved the final version of the article to be published.

## Conflict of Interest

The authors declare that the research was conducted in the absence of any commercial or financial relationships that could be construed as a potential conflict of interest.
